# Diet in Diphtheria with Special Consideration of the Proper Food for Children

**Published:** 1891-02

**Authors:** 


### Diet in Diphtheria with Special Consideration of the Proper Food for Children.

The condition of the alimentary canal of the child from birth should receive the most thoughtful attention of the mother. That which goes into it should be most carefully selected and prepared, and that which goes from it should be closely observed from time to time. At frequent intervals the child should be weighed in a balance, and if found wanting more scrupulous care should be given to the diet than before with the view of making up the deficiency. The mother should know that in the case of the child it not only eats to live, but should also eat to grow. The best and most nutritious food should be selected, it should be given at regular and proper intervals, and the child should be taught to eat carefully and slowly. From the beginning of the teething process the family dentist should be visited at regular intervals with a view to caring for the teeth, which are of greater importance to the well-being of the child than is usually appreciated by the laity. The child should be taught how to masticate, for thorough mastication needs to be taught. We help them in learning to walk, we aid them in learning to talk, and we certainly should assist them in learning to properly prepare, to properly grind their food. Lessons in mastication should be a part of the training of the infant. Mothers should study not merely how to please the palate, they should familiarize themselves with the primary principles of phsiology to the extent that a proper selection of food may be made in order to attain the completest nutrition. Variety is the spice of diet. Not only does the palate demand variety, but the tissues as well. Let us not forget to remind the mothers that the five digestive fluids are, from the beginning, dependent upon the proper activity of the digestive glands, which are a part of the glandular system, and that the entire system must be doing proper work or else the individual glandular organs will become crippled. Reasonable muscular exercise should be insisted upon, and if the child be too young to exercise himself, he should be given the benefit of massage, with daily trips in the fresh air from the very earliest weeks of infantile life. There is far less danger from taking cold in the winter time than there is from being poisoned from breathing bad air in close, ill-ventilated rooms. With the surface properly protected there is no danger from cold air. In many homes there is more sewer gas than oxygen, and much of the loss of appetite, and impaired nutrition in the child's life is due to a neglect of general and personal hygiene. The child that is properly clothed can, from the very first months of its existence, almost live out of doors the greater part of the day time, winter and summer. There can be no demand for fuel in any furnace unless there be drafts; there can be no consumption of fuel unless there is plenty of oxygen.

It goes without saying that the well-nourished, well-cared for, plant or tree can withstand the ravages of the elements better than the stunted one. This truth is illustrated among children when zymotic disease is threatening them with its ravages. The well fed child, the child that has a clamorous appetite, whose diet is well selected, whose digestion is well performed, who takes plenty of physical exercise, who lives out doors and is well clothed, breathes plenty of oxygen, for instance, rarely takes diphthera, and when he does, unless there has been dire neglect of the preliminary announcements of the disease, the case can probably be easily managed, at least in the majority of cases.

Of course the consideration of all these hygienic points of early life does good only in the direction of equipping the child in a manner to resist disease. When we are brought face to face with a case of bona-fide, well-developed diphtheria, happy are we if the child has had proper management from birth. If it has not we have all the more reason for availing ourselves of the opportunity for good which lies in the direction of nourishing the afflicted one in a manner to give him the greatest power of resistance against the diphtheritic virus. I claim that we are neglectful of our duty if we do not preach, yea, if we do not almost fanatically announce, in season and out of season, to the parent of the child under our care the importance of oxygen, and exercise, and fresh air, and urge the giving of a diet easy of digestion and assimilation, and likewise of excretion. I repeat that we should teach the mothers not only to know what goes into the child, but also carefully observe what comes out of it, and train them in the direction of recognizing variations from the normal of the various secretions of the body. The child not only runs the risk of poisoning from the sewers within our homes, but there is danger of sewerage poison from its own sewerage system, particularly if it be permitted to become clogged and gorged.

